# Pressure-pain thresholds of the plantar foot reflect relative tissue thickness and are systematically higher in active runners than non-runners

**DOI:** 10.3389/fphys.2025.1660803

**Published:** 2025-09-09

**Authors:** Claire Game, Tom Walsh, Nathan Stevenson, Werner Klingler, Scott C. Wearing

**Affiliations:** ^1^ Podiatry Professionals, Canberra, ACT, Australia; ^2^ Division of Medical Services, Princess Alexandra Hospital, Brisbane, QLD, Australia; ^3^ Brain Modelling Group, QIMR Berghofer, Brisbane, QLD, Australia; ^4^ Department of Neurosurgery, Ulm University, Ulm, Germany; ^5^ School of Medicine and Health, Technical University of Munich, Munich, Germany; ^6^ Basel Academy for Quality and Research in Medicine, Basel, Switzerland

**Keywords:** dolorimetry, algometer, pain sensitivity, foot sole, perception, connective tissue, somatosensory, biomechanics

## Abstract

**Background:**

Athletes have been shown to have greater tolerance and, to a lesser extent, a lower sensitivity to mechanical pain. However, little is known as to whether the pressure-pain sensitivity of the plantar tissues of the foot of runners, which are exposed to repeated, high-impact forces during running, differs to those of non-runners. This study evaluated topographical pressure-pain sensitivity maps of the plantar foot, and at a reference site of the palmar hand, in competitive distance runners and healthy, non-runners and explored the relationship between pressure-pain thresholds and skin and subcutaneous tissue morphology.

**Methods:**

Mechanical pressure-pain thresholds (PPTs) were measured using an algometer fitted with a cylindrical probe (1 cm^2^) in 23 competitive distance runners [mean (±SD) age, 39.7 ± 12.0 years; height, 1.75 ± 0.09 m; weight, 68.0 ± 8.4 kg] and an equivalent number of healthy non-runners [mean (±SD) age, 36.6 ± 10.1 years; height, 1.73 ± 0.10 m; weight, 77.6 ± 15.9 kg]. PPTs were determined, bilaterally, using an increasing ramp of ≈30 kPa/s at six standardised sites of the plantar foot, including the centre of the plantar calcaneal area (PCA), the Abductor Hallucis muscle belly (ABH), the plantar metatarsal area of the first (1MH), third (3MH), and fifth (5MH) metatarsal heads, the Abductor Digiti Minimi muscle belly (ADM), as well as the Abductor Pollicis Brevis muscle belly (THE) of the corresponding hand. Skin and subcutaneous tissue thickness at each site was measured using B-mode ultrasound equipped with an 18–4 MHz linear array transducer. Potential differences in PPT values and tissue thickness between groups were assessed using three-way repeated-measures ANOVA and pairwise comparisons with Šidák’s adjustment for multiple comparisons. Relationships between measures of PPT and tissue thickness were explored using nonlinear regression with skin and subcutaneous tissue thickness as the independent variable. Akiake’s Information Criterion was used to assess logit and polynomial fits (linear, quadratic and cubic).

**Results:**

Mean PPT values in runners were, on average, 24% higher than those of non-runners, across all sites (F_1,43_ = 4.6, P = 0.038). Pain sensitivity varied significantly across the plantar surface of the foot in both runners and non-runners (F_3.2_, _139.9_ = 82.5, P <0 .001). PPTs at the PCA were significantly higher (range, 18.6–31.7 kPa) and the ABH significantly lower (range, −31.7 − −6.2 kPa) than those at all other foot sites (P < 0.05). Similarly, mean PPT measured at the THE was significantly lower than that measured at all plantar foot sites (range, −36.9 − −5.1 kPa) in both groups. Runners also presented with significantly thinner tissues than non-runners (F_4, 177_ = 14.1, P = 0.016) at the PCA [−1.5 mm (−2.8, −0.2), P <0 .05], 1MH [−1.0 mm (−2.0, −0.1), P <0 .05], and ADM [−1.4 mm (−2.6, −0.2), P <0 .05]. The relationship between PPT and tissue thickness was best described by a logit function in runners and non-runners (range R^2^, 88%–95%). Normalization of pedal PPT values to those of the hand, mitigated the bias in plantar foot PPTs between groups, without altering the shape of the logit function.

**Conclusion:**

Distance runners presented with lowered sensitivity to mechanical pain than non-runners, despite relatively thinner plantar foot tissues. The topographical variation in PPTs across the plantar foot can be effectively modeled as a function of relative plantar tissue thickness, and the hypoalgesic bias in runners may be mitigated by the normalization of PPT values to those of the hand, without altering the shape of the logit function. Hence, centrally-mediated pathways may underpin the mechanical hypoalgesia of the plantar foot in runners.

## 1 Introduction

Pressure dolorimetry is commonly used as part of established quantitative sensory testing protocols to mechanically induce deep tissue pain and measure mechanical pain sensitivity (Graven-Nielsen, 2022; Rolke et al., 2006). Deep pressure-pain thresholds (PPTs), which involve the blunt mechanical indentation of skin and subcutaneous tissues, reflect the lowest principal stress that first elicits pain (Fischer, 1987), and are classically thought to be mostly mediated by low-threshold mechanoreceptors of thinly-myelinated A-delta fibres and, to a lesser extent C-fibers, via the anterior spinothalamic tract (Rolke et al., 2006; Yam et al., 2018; Simone et al., 1994). More recently, high-threshold, polymodal receptors of large-diameter, thickly-myelinated, A-delta fibers (Fleckenstein et al., 2017; Nagi et al., 2019), primarily located deep within the superficial fascia and the deep fascial tissues (Case et al., 2021), have also been implicated. PPTs evaluating discrete sites of the foot have been reported within the literature for over half a century, and have most commonly been used as remote test sites to aid in the identification of widespread mechanical hyperalgesia (Weidenbacker et al., 1963; Brennum et al., 1989; Rolke et al., 2005; Graven-Nielsen et al., 2010). Discrete PPTs of the plantar foot are well known to be higher than those of the palmar hand, presumably reflecting the lower density and higher activation thresholds of mechanoreceptors of the distal lower extremity (Rolke et al., 2005; Ro et al., 2006; Kennedy and Inglis, 2002; Strzalkowski et al., 2018; Corniani and Saal, 2020).

More recently, topographical pressure-pain sensitivity maps of the plantar aspect of the feet have become an area of increased research interest, particularly within the context of athletic footwear research (Hodge et al., 2009; Xiong et al., 2011; Xiong et al., 2013; Weerasinghe et al., 2016; Tornero-Caballero et al., 2016; Wu et al., 2024; Madeleine et al., 2014). Such maps have reinforced the concept that afferent innervation and pain sensitivity in healthy adults varies across the sole of the foot (Strzalkowski et al., 2018), with higher pain thresholds and lower sensitivity commonly reported beneath the heel and plantar metatarsal area (Hodge et al., 2009; Xiong et al., 2011; Xiong et al., 2013; Weerasinghe et al., 2016; Tornero-Caballero et al., 2016; Wu et al., 2024; Madeleine et al., 2014). Purported to reflect, in part, the morphology or mechanical properties of the skin and subcutaneous tissues (Kennedy and Inglis, 2002; Xiong et al., 2013; Weerasinghe et al., 2016; Rodrigo et al., 2013; Strzalkowski et al., 2015a; Katic et al., 2022), these sites have also been shown to experience the highest principal stress on the foot during walking and running (Wearing et al., 2001; Hong et al., 2012; Hohmann et al., 2016). It is surprising, therefore, that minimal research has evaluated whether pressure-pain sensitivity maps of the feet of distance runners, in which the plantar tissues are exposed to repeated impacts associated with foot strike, differ to those of non-runners. It is particularly surprising, given that aerobic exercise has been shown to induce an acute, but transitory, hypoalgesia in healthy adults (Tomschi et al., 2024), and that athletes have often been shown to have moderately higher PPTs and, therefore, lower sensitivity to mechanical pain, than non-athletes across a wide variety of body sites (Thornton et al., 2024; Pacheco-B et al., 2020). However, as noted in a recent systematic review, which included 17 studies, involving 1,397 athletes, there is a need to evaluate pain sensitivity in specific athletic groups given the considerable heterogeneity in mechanical PPTs observed across different athlete cohorts (Thornton et al., 2024).

Consequently, this study aimed to evaluate whether pressure-pain sensitivity of the plantar surface of the foot, and a remote site of the palmar hand, differed in healthy, competitive distance runners compared to non-runners. Specifically, we tested the null hypothesis that there would be no difference in pressure pain thresholds across the plantar aspect of the foot and palmar hand of competitive runners and non-runners. We also evaluated whether PPT values could be effectively modelled as a function of relative plantar tissue thickness in each group.

## 2 Materials and methods

### 2.1 Participants

Participants were recruited over an 8-month period. Advertisements for volunteers were placed in running and triathlon clubs across the greater metropolitan area including online advertising and flyers distributed throughout the University faculty and students. In accordance with established criteria, participants were classified as runners if they self-reported that; (a) they trained with a purpose of performance enhancement and to compete, (b) partook in regular endurance running training at least three times per week, and (c) were formally registered with a regional or national sport federation (McKay et al., 2022; MacMahon and Parrington, 2017). The latter two criteria were also confirmed independently by evaluation of training log books and federation registers by a member of the research team. Non-runners, in contrast, were active individuals who did not participate in activities where running was a primary focus, did not participate in running-related competitions, and did not run for competitive performance enhancement.

As there is currently no published data regarding PPT values involving the plantar tissues of the foot in runners and non-runners to guide a sample size calculation, the study sample size was calculated *a priori* based on previously published PPT values reported for plantar foot sites in healthy, middle-aged adults recruited from the general population (Ríos-León et al., 2019). A sample size of 22 runners, and an equivalent number of non-runners, was estimated to have sufficient statistical power (β = 0.10) at an alpha (α) level of 0.05 to identify the minimal detectable change in PPT (98 kPa) reported for the plantar calcaneal area (Nagi et al., 2019). Hence, the study was not only statistically powered to identify mean differences in PPT values reported in the literature for individuals with and without plantar heel pain (Ríos-León et al., 2019; Fernandez-Lao et al., 2016), but also for locally reported changes in PPTs following high-intensity exercise (Tomschi et al., 2024). The project was undertaken in accordance with the principles of the Declaration of Helsinki. Written consent was obtained from all study participants after a verbal and written explanation of the project as per the requirements of the University Human Research Ethics Committee clearance.

### 2.2 Protocol

All participants presented to thermoneutral laboratory wearing lightweight comfortable clothing and having abstained from vigorous physical activity, caffeinated beverages and the use of analgesics or muscle relaxants within the previous 24 h. Measurements of body height (stretch stature) were made to the nearest millimeter, using a Harpenden stadiometer (Cranlea and Co, Birmingham, UK), and body weight was recorded to the nearest 0.1 kg using a digital scale (8W8600, Tanita, Tokyo, Japan). The Patient-Reported Outcome Measurement Information System (PROMIS) Profile‐29 was used to assess six domains of physical, mental and social health, including Physical Function, Anxiety, Depression, Fatigue, Sleep Disturbance, and Social Dysfunction. The self-reported PROMIS questionnaire contained four items for each domain and raw scores for each domain were standardized and expressed as t-scores with a population mean of 50 and a standard deviation of 10 (Schalet et al., 2016). Thus, the higher the numerical score for a given domain, the more of the attribute that was measured (Haupt et al., 2023).

#### 2.2.1 Mechanical pressure-pain thresholds (PPTs)

**FIGURE 1:**
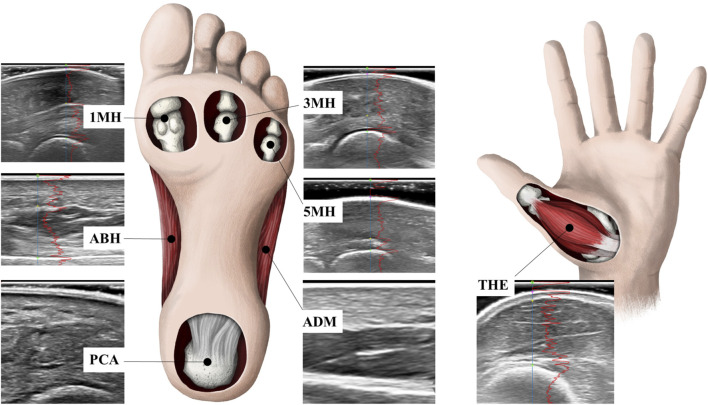
Pressure-pain thresholds and skin and subcutaneous tissue thickness were determined bilaterally at standardised sites including the centre of the plantar calcaneal area (PCA), the Abductor Hallucis muscle belly (ABH), the plantar metatarsal area of the first (1MH), third (3MH), and fifth (5MH) metatarsal heads, the Abductor Digiti Minimi muscle belly (ADM), as well as the Abductor Pollicis Brevis muscle belly (THE) of the corresponding hand.

Topographical pressure-pain sensitivity was measured bilaterally, over seven locations, by a single, trained operator using standardised instructions and according to the methods outlined by Ríos-León et al. (2019). In brief, a single operator manually applied pressure, perpendicular to the skin, at each measurement site using a customised electronic algometer (FDIX-25, Wagner Instruments, Greenwich, CT, United States). The load cell of the algometer had a full-scale of 112 N and resolution of 0.05 N, and was fitted with a cylindrical polyurethane probe (Ø, 11 mm), with a contact area of 1 cm^2^. The algometer was modified to provide the operator with a real-time visual display of the applied force and was fitted with an external mechanical trigger that enabled participants to stop the acquisition of data once they perceived the applied pressure first changed to pain. The algometer was calibrated prior to data collection. Pressure-pain thresholds were determined bilaterally at seven sites; six involving the plantar surface of the foot and one site involving the palmar surface of the hand (Figure 1). To aid comparison to previous research (Tornero-Caballero et al., 2016; Ríos-León et al., 2019; Fernandez-Lao et al., 2016), standardised sites of the foot included the centre of the plantar calcaneal area (PCA), the Abductor Hallucis muscle belly (ABH), the plantar metatarsal area of the first (1MH), third (3MH), and fifth (5MH) metatarsal heads, and the Abductor Digiti Minimi muscle belly (ADM). The Abductor Pollicis Brevis muscle belly (THE) of the corresponding hand was also evaluated (Ríos-León et al., 2019). The THE was specifically selected as a remote test site to evaluate potential widespread effects, as it is innervated by the median nerve (T1) rather than lumbosacral plexus which innervates the plantar tissues of the foot.

Participants were positioned prone, with the knee and ankle of the test limb flexed to 45° and the dorsal surface of the foot and ventral surface of the hand resting on a rigid support surface. Prior to testing, the method was first demonstrated at a non-test site involving the arm and plantar foot to familiarize participants with the protocol. The pressure pain threshold (PPT) was subsequently determined at each site using two series of ascending stimulus intensities, each manually applied to match a slowly increasing ramp of 30 kPa/s which was shown on a built-in visual display. Participants were blinded to the display and requested to press the trigger to cease the test once the sensation of pressure first changed to pain (Fischer, 1987; Saban and Masharawi, 2016). There was no upper pressure limit applied by the operator during testing and the order of testing was randomized between sites and counterbalanced between limbs. A minimum rest period of 30-s was provided between trials in order to minimize potential temporal summation (Nie et al., 2005). Force data were sampled at a rate of 100 Hz and the peak pressure calculated. The mean PPT from two trials was calculated at each site and used for later analysis. The technical error of measurement for repeated measures of PPT and loading rate determined across all sites and in both limbs in the current study was 76.3 ± 21.1 kPa and 1.8 ± 0.5 kPa/s, respectively. As calculated in the current study, the technical error of measurement is identical to that of the “within-subject standard deviation” popularized by Bland and Altman (Bland and Altman, 1996) and is often interpreted as the typical range of measurement error that can be expected to occur with repeated measurement (Harris and Smith, 2009). According to Saban and Masharawi (2016) and others (Walton et al., 2011), the minimal detectable change in PPT values for the plantar calcaneal area and ventral shank are in the order of 98–161 kPa. Given that the normalization of PPT values to those of remote sites tends to show greater sensitivity and less temporal drift than absolute values (Rolke et al., 2005; Kosek et al., 1993; Fredriksson et al., 2000), PPT values at each site were also normalized to those of the THE of the corresponding hand and expressed as a proportion.

#### 2.2.2 Skin and subcutaneous tissue thickness

Blinded ultrasound examination of each site was undertaken by an experienced operator using a high-resolution B-mode ultrasound machine (iU22, Philips Medical Systems, Bothell, WA, United States), equipped with an 18–4 MHz linear array transducer and a standardized protocol. Ultrasonic examination of the unloaded skin and subcutaneous tissues was undertaken using a modified method to that outlined by Lin et al. (2015). Participants were positioned prone with a neutral ankle and the knee flexed at right angles (Maemichi et al., 2020). Each site was initially imaged transversely and axially in dynamic mode to allow active movement and to aid identification of key structures, including muscle tendon, epitendinous and paratendinous structures. Each location was subsequently marked using an indelible skin marker to ensure consistent placement for ultrasound and PPT testing. The ultrasound transducer was then positioned over the center of the measurement site, coincident with the long axis of the foot. Sagittal images were acquired using a thick layer of acoustic coupling gel, such that the site of measurement was located at the center of the image. Care was taken to ensure the transducer did not touch the plantar surface of the foot or compress the plantar fibro-adipose tissues (Lin et al., 2015). Only images in which acoustic coupling gel could be clearly visualized between the transducer and the surface of the skin were stored for later analysis. Up to eight replicate images were acquired for all structures, with the order of imaging counterbalanced between limbs and sites. All ultrasound images were subsequently exported in DICOM format to PC for post processing. The thickness of the skin and subcutaneous tissue at each site was analyzed using custom, semi-automated MATLAB software (MathWorks Inc., Natick, MA) with the aid of a grayscale profile. All measurements were undertaken by a second operator and in a blinded manner. The technical error of measurement for repeated measures of tissue thickness at all sites was less than 1.0%.

### 2.3 Statistical analysis

Statistical analysis was performed using IBM-SPSS statistical software (version 26, IBM Corp. Armonk, NY, United States). All data were evaluated for normality using the Shapiro Wilk’s test. Outcome variables were determined to be normally distributed and hence, means and standard deviations have been used as summary statistics. The homogeneity of variances was assessed using Levene’s statistic. Potential differences in age and anthropometric characteristics and patient reported outcome measures of health-related quality of life (PROMIS subscales) between runners and non-runners were analyzed using independent t-tests. Differences in sex-distribution between groups was examined using Fischer’s exact test. Potential differences in absolute and normalized PPT values and tissue thickness were assessed using three-way repeated-measures ANOVA within a generalized linear modelling framework. In each case, group (runners and non-runners) was treated as a between–subject factor, while limb (left and right) and site (PCA, ABH, 1MH, 3MH, 5MH, ADM, and THE) were treated as within–subject factors. Underlying assumptions regarding the uniformity of the variance–covariance matrix were assessed using Mauchly’s test of sphericity. When the assumption of uniformity was violated, an adjustment to the degrees of freedom of the F ratio was made using Greenhouse–Geisser Epsilon, thereby making the F-test more conservative. Significant effects were subsequently investigated using pairwise comparisons with Šidák’s adjustment for multiple comparisons. Potential relationships among measures of PPT, tissue thickness, anthropometric characteristics and measures of health-related quality of life were investigated using Pearson correlations. Relationships among measures of absolute and normalized PPT and tissue thickness were further explored graphically, and, subsequently, a logit function was fit to the data using nonlinear regression with skin and subcutaneous tissue thickness as the independent variable (x) and PPT as the dependent variable (y). The logit functional form fit to the data is defined as,

$$y=f\left(x\right)=\beta_{1}\ln\left(\frac{x}{\beta_{2}-x}\right)+\beta_{3}$$

where $\beta_{1}$ effectively represents the overall gain of the pressure-pain response, ln is the natural logarithm of relative tissue thickness, which defines the curvilinear nature of the relationship between pressure-pain response and tissue thickness, $\beta_{2}$ is the maximum skin and subcutaneous tissue thickness, and $\beta_{3}$ reflects the pressure-pain offset. The selection of the logit function was assessed by comparing the Akiake’s Information Criterion (AIC) to polynomial fits (linear, quadratic and cubic) and the functional fit with the minimum AIC was selected. The goodness of fit was measured using the r-squared coefficient between values predicted by the fitted logit function and observed data. In all cases, an alpha level of 0.05 was used for overall tests of significance.

## 3 Results

### 3.1 Participant characteristics and health-related quality of life

Table 1 summarises the demographic characteristics and health-related quality of life of healthy runners and non-runners. As anticipated, non-runners were significantly heavier than competitive runners (t_33_ = 2.6, P = 0.015), and presented with a significantly higher body mass index (BMI) (t_26_ = 3.6, P = 0.002). According to World Health Organisation guidelines, all trained runners were within a “normal weight” range, while non-runners ranged from “normal weight” through to “Obese Class II” (WHO Expert Consultation, 2004). There was no significant difference between groups for patient reported outcome measures of health-related quality of life, with the exception of anxiety, which was significantly lower in runners than non-runners (t_42_ = 2.69, P = 0.010).

**TABLE 1:** Characteristics of healthy, trained runners and non-runners.

	Non-runners	Runners
n	23	23
Female/Male	9/14	9/14
Age (years)	36.6	39.7
	(10.1)	(12.0)
Height (m)	1.73	1.75
	(0.10)	(0.09)
Mass (kg)	77.6	68.0^a^
	(15.9)	(8.4)
Body Mass Index (kg/m^2^)	26.0	22.2^a^
	(4.9)	(1.5)
PROMIS Physical Function	56.0	56.6
	(2.9)	(1.7)
PROMIS Anxiety	50.7	45.3^a^
	(7.6)	(5.9)
PROMIS Depression	45.3	42.8
	(7.2)	(3.8)
PROMIS Fatigue	49.9	47.4
	(8.5)	(6.0)
PROMIS Sleep disturbance	48.6	46.5
	(6.7)	(6.1)
PROMIS Social dysfunction	58.4	59.6
	(5.8)	(6.3)

PROMIS, Patient-Reported Outcome Measurement Information System assessed self-reported Physical Function, Anxiety, Depression, Fatigue, Sleep Disturbance, and Social Dysfunction.

^a^ Statistically significant difference between groups (P < 0.05).

### 3.2 Skin and subcutaneous tissue thickness

Runners presented with significantly thinner skin and subcutaneous tissues than non-runners across all sites [mean difference (95%CI) = −0.8 mm (−1.5, −0.0), P < 0.05]. As expected, there was also a significant main effect of measurement site on tissue thickness (F_4, 177_ = 1,005.1, P < 0.001). Mean skin and subcutaneous tissue thickness was greatest at the PCA [group mean (95%CI) = 16.4 mm (15.8, 17.1)] and thinnest at the THE [group mean (95%CI) = 1.9 mm (1.8, 2.1)]. Post hoc analysis revealed that, irrespective of group, skin and subcutaneous tissue thickness was significantly different between all sites (P < 0.001). The ANOVA model also revealed a significant group by site interaction (F_4, 177_ = 14.1, P = 0.016), whereby runners presented with significantly thinner tissues at the PCA [mean difference (95%CI) = −1.5 mm (−2.8, −0.2), P < 0.05], 1MH [mean difference (95%CI) = −1.0 mm (−2.0, −0.1), P < 0.05], and ADM [mean difference [95%CI) = −1.4 mm (−2.6, −0.2), P < 0.05]. There was no significant main effect for limb or interaction effects between group and limb or limb and site.

Pearson correlations among patient characteristics and subcutaneous tissue thickness at each site in runners and non-runners are summarised in Tables 2, 3. Plantar tissue thickness in non-runners tended to be positively, though modestly, correlated with body mass at all foot sites (P < 0.05) and with BMI at all foot sites (P < 0.05), except for 3MH and 5MH. In contrast, tissue thickness in runners was not significantly correlated with BMI at any site, but rather was positively associated with both body mass and body height across all foot sites (P <0.05), except 1MH. The thickness of the skin and subcutaneous tissues in runners was also negatively correlated with age at the PCA, ADM and 5MH, bilaterally (P < 0.05). Pearson correlations revealed no significant linear relationships among tissue thickness and health-related quality of life variables in either group.

**TABLE 2:** Correlation coefficients (P value) among participant characteristics and plantar skin and subcutaneous tissue thickness at sites of the left foot in runners and non-runners (n = 23).

	Age (years)	Height (m)	Weight (kg)	BMI (kg/m^2^)
Non-runners				
Plantar Calcaneal Area (PCA)	0.07	0.06	0.51^a^	0.53^a^
	(0.756)	(0.786)	(0.014)	(0.010)
Abductor Hallucis Muscle (ABH)	0.24	−0.31	0.71^a^	0.59^a^
	(0.275)	(-0.153)	(0.000)	(0.003)
First Metatarsal Head (1MH)	0.22	0.32	0.56^a^	0.41
	(0.304)	(0.142)	(0.005)	(0.050)
Third Metatarsal Head (3MH)	0.04	0.39	0.53^a^	0.35
	(0.870)	(0.067)	(0.009)	(0.098)
Fifth Metatarsal Head (5MH)	−0.11	0.39	0.53^a^	0.34
	(0.620)	(0.068)	(0.010)	(0.111)
Adductor Digiti Minimi Muscle (ADM)	0.00	0.23	0.51^a^	0.44^a^
	(0.999)	(0.287)	(0.013)	(0.035)
Abductor Pollicis Brevis muscle (THE)	0.27	0.31	0.70^a^	0.58^a^
	(0.208)	(0.151)	(0.000)	(0.004)
Runners				
Plantar Calcaneal Area (PCA)	−0.51^a^	0.56^a^	0.61^a^	0.32
	(0.013)	(0.005)	(0.002)	(0.131)
Abductor Hallucis Muscle (ABH)	−0.29	0.49^a^	0.51^a^	0.19
	(0.186)	(0.018)	(0.013)	(0.385)
First Metatarsal Head (1MH)	−0.19	0.24	0.19	−0.02
	(0.394)	(0.262)	(0.397)	(0.912)
Third Metatarsal Head (3MH)	−0.27	0.48^a^	0.44^a^	0.10
	(0.206)	(0.019)	(0.035)	(0.652)
Fifth Metatarsal Head (5MH)	−0.48^a^	0.67^a^	0.59^a^	0.10
	(0.019)	(0.000)	(0.003)	(0.638)
Adductor Digiti Minimi Muscle (ADM)	−0.68^a^	0.50^a^	0.57^a^	0.33
	(0.000)	(0.016)	(0.004)	(0.129)
Abductor Pollicis Brevis muscle (THE)	−0.27	0.32	0.28	0.06
	(0.212)	(0.140)	(0.202)	(0.798)

^a^ Indicates a statistically significant association (P < .05). BMI, body mass index.

**TABLE 3:** Correlation coefficients (P value) among participant characteristics and plantar skin and subcutaneous tissue thickness at sites of the right foot in runners and non-runners (n = 23).

	Age (years)	Height (m)	Weight (kg)	BMI (kg/m^2^)
Non-runners				
Plantar Calcaneal Area (PCA)	−0.01	0.09	0.53^a^	0.53^a^
	(0.981)	(0.689)	(0.010)	(0.009)
Abductor Hallucis Muscle (ABH)	0.31	0.36	0.52^a^	0.47^a^
	(0.147)	(0.094)	(0.011)	(0.022)
First Metatarsal Head (1MH)	0.06	0.29	0.53^a^	0.50^a^
	(0.781)	(0.181)	(0.009)	(0.015)
Third Metatarsal Head (3MH)	0.05	0.38	0.50^a^	0.33
	(0.835)	(0.072)	(0.014)	(0.129)
Fifth Metatarsal Head (5MH)	−0.09	0.26	0.36	0.22
	(0.684)	(0.224)	(0.094)	(0.312)
Adductor Digiti Minimi Muscle (ADM)	−0.04	0.35	0.58^a^	0.45^a^
	(0.858)	(0.104)	(0.003)	(0.030)
Abductor Pollicis Brevis muscle (THE)	0.11	0.06	0.54^a^	0.55^a^
	(0.632)	(0.774)	(0.008)	(0.007)
Runners				
Plantar Calcaneal Area (PCA)	−0.42^a^	0.59^a^	0.70^a^	0.33
	(0.044)	(0.003)	(0.000)	(0.120)
Abductor Hallucis Muscle (ABH)	−0.06	0.47^a^	0.44^a^	0.11
	(0.787)	(0.023)	(0.037)	(0.603)
First Metatarsal Head (1MH)	−0.11	0.20	0.29	0.21
	(0.624)	(0.352)	(0.177)	(0.326)
Third Metatarsal Head (3MH)	−0.27	0.45^a^	0.41^a^	0.10
	(0.214)	(0.031)	(0.052)	(0.661)
Fifth Metatarsal Head (5MH)	−0.42^a^	0.43^a^	0.43^a^	0.18
	(0.047)	(0.042)	(0.040)	(0.407)
Adductor Digiti Minimi Muscle (ADM)	−0.65	0.53^a^	0.59^a^	0.32
	(0.001)^a^	(0.010)	(0.003)	(0.143)
Abductor Pollicis Brevis muscle (THE)	0.11	0.21	0.28	0.20
	(0.616)	(0.345)	(0.202)	(0.356)

^a^ Indicates a statistically significant association (P < .05). BMI, body mass index.

### 3.3 Mechanical pressure-pain thresholds

The mean loading rate across all groups, limbs and sites was 27 ± 1 kPa/s. Table 4 shows mean PPT values measured at the hand (THE) and plantar foot in runners and non-runners. The ANOVA model demonstrated a significant main effect for group on PPT values (F_1,43_ = 4.6, P = 0.038). Post hoc analysis revealed that mean PPT values of runners were, on average, 24% higher than those of non-runners across all sites [mean difference (95%CI) = 90.0 kPa (5.1, 174.8), P < 0 .05]. There was also a significant main effect for site on PPT values (F_3.2_, 139.9 = 82.5, P < 0.001). The mean PPT measured at the THE was significantly lower than that measured at all plantar foot sites (range, −36.9 − −5.1 kPa), irrespective of group. In considering only plantar sites of the foot, the mean PPT determined at the ABH was also significantly lower (range, −31.7 − −6.2 kPa) across groups, and the PCA significantly higher (range, 18.6–31.7 kPa), than that measured at all other foot sites (P < 0.05). Mean PPTs determined at the 1MH were also significantly lower than those measured at 3MH [mean difference (95%CI) = −5.1 kPa (−8.8, −1.4)], 5MH [mean difference (95%CI) = −7.0 kPa (−12.4 − −1.7)], and ADM [mean difference (95%CI) = −6.1 kPa (−11.8, −0.4)]. There were no significant between-site differences in PPT values measured at 3MH, 5MH and ADM. Similarly, there was no significant main effect for limb nor interaction effects between group, limb and site.

**TABLE 4:** Mean (SD) pressure-pain threshold values (kPa) for runners and non-runners.

PPT	Non-runners		Runners^a^	
	Left	Right	Left	Right
n	23	23	23	23
Plantar Calcaneal Area (PCA)^b^	559.6	564.0	690.7	704.2
	(210.6)	(194.2)	(260.6)	(282.8)
Abductor Hallucis Muscle (ABH)^b^	285.0	305.3	364.1	355.0
	(104.2)	(116.2)	(150.1)	(146.7)
First Metatarsal Head (1MH)^b^	343.5	343.4	409.1	459.5
	(119.6)	(123.0)	(183.1)	(158.7)
Third Metatarsal Head (3MH)	395.1	396.7	490.7	476.2
	(142.6)	(142.4)	(193.3)	(206.5)
Fifth Metatarsal Head (5MH)	421.2	402.1	526.0	487.1
	(150.7)	(156.2)	(260.2)	(168.7)
Adductor Digiti Minimi Muscle (ADM)	417.7	424.8	491.7	465.7
	(166.1)	(168.3)	(190.9)	(178.6)
Abductor Pollicis Brevis muscle (THE)^b^	231.3	251.5	307.7	313.1
	(73.5)	(92.1)	(91.4)	(100.1)

PPT, Pressure-pain threshold.

^a^ Significant main effect for group (P < .05).

^b^ Significantly different from all other sites (P < .05).

**FIGURE 2:**
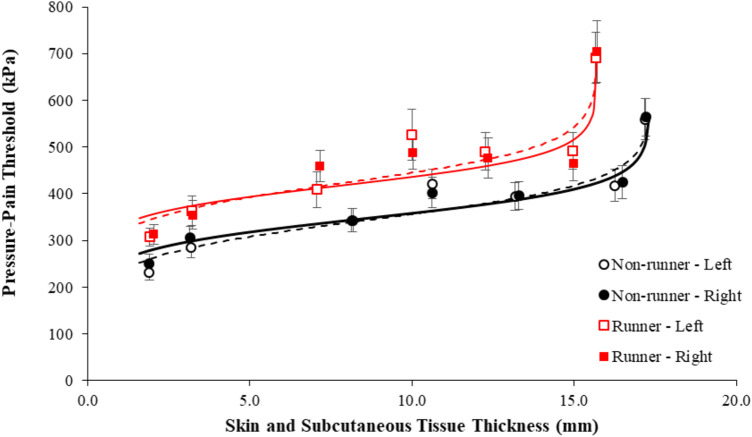
Mean pressure-pain thresholds of the plantar sole of the left (dashed line) and right foot (solid line) in active runners (red shading) and non-runners (black shading) presented as a function of the thickness of the skin and subcutaneous tissues. Error bars represent standard error. Data was best fit by a logit model (R^2^, 88%–95%).

Figure 2 demonstrates mean PPT values in each limb of runners and non-runners as a function of skin and subcutaneous tissue thickness. In general, the data were best fit by the logit function, which had the lowest AIC compared to polynomial fits and R^2^ values of 88%–95% (Table 5). The logit function supports the observed findings with a heightened β3 coefficient (elevated PPT) and lower β2 coefficient (reduced maximum skin and subcutaneous tissue thickness) in runners than non-runners.

**TABLE 5:** Akiake’s Information Criterion (AIC) for logit and polynomial fits of pressure-pain thresholds in limbs of runners and non-runners as a function of tissue thickness. Coefficients and r-squared values for goodness of the logit are also shown.

	Non-runners		Runners	
AIC functional fit	Left	Right	Left	Right
Logit	51.42	49.80	55.15	60.15
Linear	52.28	52.78	56.32	58.89
Quadratic	61.69	61.83	65.72	68.80
Cubic	87.93	88.84	93.74	95.89
Logit Fit				
R^2^	0.91	0.95	0.88	0.89
β1	37.63	32.22	37.23	31.02
β2	17.22	17.22	15.67	15.70
β3	332.01	340.42	406.71	408.24

With the exception of fatigue, which was modestly, though negatively, correlated with PPTs at the PCA of both the left (r = −0.45, P < 0.030) and right (r = −0.47, P < 0.022) feet of runners, univariate correlations revealed no other significant associations among PPTs, anthropometric variables and self-reported health-related quality of life in either group.

### 3.4 Normalized pressure-pain thresholds

Table 6 shows PPT values at plantar foot sites following normalization to PPT values at the hand (THE) in runners and non-runners. In contrast to raw PPT values, there was no significant main effect for group on normalized PPT values. There was, however, a significant main effect for site on normalized PPT values (F_3, 132_ = 73.9, P < 0.001). Differences in normalized PPT showed the same pattern between sites as raw PPT values. Mean normalized PPT values at the ABH were significantly lower (range, −1.20 − −0.24), and those at the PCA significantly higher (range, 1.20–0.70), than that measured at all other foot sites (P < 0.05). Normalized PPTs determined at the 1MH were also significantly lower than those measured at 3MH [mean difference (95%CI) = −0.19 (−0.33, −0.04), P < 0.05], 5MH [mean difference (95%CI) = −0.26 (−0.45 − −0.08), P < 0.05], and ADM [mean difference (95%CI) = −0.25 (−0.44, −0.06), P < 0.05]. There were no significant between-site differences in normalized PPT values measured at 3MH, 5MH and ADM. Similarly, there was no significant main effect for limb or interaction effects between group, limb and site.

**TABLE 6:** Pressure-pain threshold values normalized to hand values in runners and non-runners.

nPPT	Non-runners		Runners	
	Left	Right	Left	Right
n	23	23	23	23
Plantar Calcaneal Area (PCA)^a^	2.47	2.31	2.31	2.49
	(0.77)	(0.59)	(0.82)	(0.98)
Abductor Hallucis Muscle (ABH)^a^	1.24	1.25	1.18	1.15
	(0.30)	(0.38)	(0.35)	(0.38)
First Metatarsal Head (1MH)^a^	1.53	1.42	1.33	1.51
	(0.47)	(0.40)	(0.49)	(0.44)
Third Metatarsal Head (3MH)	1.73	1.61	1.64	1.55
	(0.44)	(0.41)	(0.61)	(0.58)
Fifth Metatarsal Head (5MH)	1.86	1.67	1.72	1.61
	(0.56)	(0.53)	(0.70)	(0.47)
Adductor Digiti Minimi Muscle (ADM)	1.84	1.76	1.64	1.52
	(0.55)	(0.53)	(0.59)	(0.46)

nPPT, normalized pressure-pain threshold.

^a^ Significantly different from all other sites (P < .05).

**FIGURE 3:**
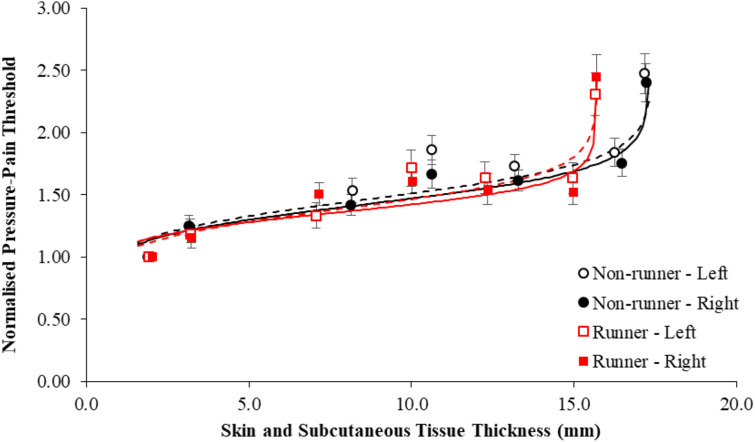
Mean pressure-pain thresholds of the plantar sole of the left (dashed line) and right foot (solid line) normalized to those of the hand in active runners (red shading) and non-runners (black shading) and presented as a function of the thickness of the skin and subcutaneous tissues. Error bars represent standard error. Data was best fit by a logit model (R^2^, 53%–69%).

Figure 3 shows normalized PPT values in each group as a function of skin and subcutaneous tissue thickness. Normalization of PPTs of the plantar foot to those of the THE reduced the overall accuracy of the fit (R^2^ range, 53%–69%) and, as expected, primarily affected the β1, and to a lesser extent, β3 coefficients, which govern the overall gain and offset of the fit, respectively. There was negligible effect of normalization of PPTs on the β2 coefficient (Table 7).

**TABLE 7:** Akiake’s Information Criterion (AIC) for logit and polynomial fits of normalized pressure-pain thresholds as a function of tissue thickness in limbs of runners and non-runners. Coefficients and r-squared values for goodness of the logit are also shown.

	Non-runners		Runners	
AIC functional fit	Left	Right	Left	Right
Logit	−12.78	−15.17	−12.75	−7.89
Linear	−12.62	−11.48	−12.09	−7.60
Quadratic	−3.07	−2.42	−2.74	2.22
Cubic	22.72	24.01	25.56	29.20
Logit Fit				
R^2^	0.69	0.60	0.61	0.53
β1	0.17	0.14	0.13	0.11
β2	17.22	17.22	15.67	15.69
β3	1.46	1.39	1.34	1.33

There were no significant correlations among normalized PPTs and anthropometric or health-related quality of life variables at any site in either group.

## 4 Discussion

This study evaluated the mechanical pressure-pain sensitivity of the plantar surface of the foot in competitive distance runners and non-runners. Mean PPT values at plantar foot sites in both groups span the broad range of values (273–947 kPa) cited for healthy adults measured using probes of the same contact area and with comparable loading rates (Hodge et al., 2009; Tornero-Caballero et al., 2016; Wu et al., 2024; Madeleine et al., 2014; Ríos-León et al., 2019). However, despite significantly thinner plantar subcutaneous tissues, trained distance runners were found to have systematically higher PPTs, bilaterally, and hence lower mechanical pain sensitivity, at all sites of the plantar foot, and thenar eminence of the hand, than non-runners. The standardized mean difference in PPTs between runners and non-runners in the current study (Hedge’s g = 0.76), reflects a moderate-to-large effect and is comparable to the magnitude of the widespread hypoalgesia (Hedge’s g, 0.40–0.69) reported previously in meta-analyses involving a wide range of athletes, across a broad range of test sites (Thornton et al., 2024; Tesarz et al., 2012). Although beyond the scope of the current study, the mechanism underpinning the widespread mechanical hypoalgesia in athletes is not entirely understood. Physical, physiological and psychological factors have been suggested to influence PPTs in athletes (Thornton et al., 2024; Lemley et al., 2015), including exercise induced hypoalgesia, in which both heightened pain inhibitory and lowered pain facilitatory pathways have been commonly, though variably, implicated using condition-pain modulation and temporal-summation paradigms, respectively (Vaegter and Jones, 2020). As noted by Vaegter et al. (2020); Vaegter et al. (2014), hypoalgsia induced by exercise and the condition-pain modulation paradigm may reflect opioidergic as well as nonopioidergic mechanisms, such as arterial baroreceptor inhibition (Ring et al., 2008), as well as altered psychological states (Pettersen et al., 2020), the recruitment of high threshold motor units (Hoeger Bement et al., 2008), and/or activation of the primary motor cortex (Jin et al., 2023). Indeed, research involving functional magnetic resonance imaging and electroencephalography has shown differences in the neural processing of nociceptive information between endurance athletes and non-athletes (Ge et al., 2021; Anders et al., 2023), while conditioned pain modulation protocols typically suggest athletes may have more effective endogenous inhibition (Flood et al., 2017; Geisler et al., 2020). Hence centrally, rather than peripherally, mediated pathways are currently thought to underpin mechanical hypoalgesia in athletes. The finding that topographical pressure-pain sensitivity in the feet and hands is systematically heightened in healthy runners in the current study lends further support to current dogma. However, further research involving temporal summation and condition pain modulation paradigms is needed to ascertain the potential contribution of facilitatory and inhibitory pathways to the widespread hypoalgesia observed at the plantar foot in runners observed in the current study.

In considering alternate explanations that may underpin the widespread mechanical hypoalgesia observed in runners in the current study, it is important to note that runners were found to differ with respect to non-runners in at least two key ways. First, runners in the current study reported significantly lower levels of anxiety, as determined by the PROMIS-29, than non-runners. The PROMIS-29 uses standardised T-scores, indicating that, in the current study, runners had lower levels of anxiety than the general population. The mean difference between groups exceeded the minimally important difference (4 points) reported for the questionnaire (Kroenke et al., 2019). Hence, the lower levels of anxiety in runners are likely to be clinically meaningful. Regular physical activity, including running, has been shown to improve mood states, and lower anxiety in healthy populations and in certain groups with chronic-pain (Oswald et al., 2020; Pereira et al., 2021; Bustamante et al., 2025; Wu et al., 2025). There is even evidence, that amongst runners, those who participate in a similar training profile to that of runners in the current study (regular competition in 10 km races and training at least 3 days a week), have the lowest scores for cognitive and somatic anxiety of all runners (Prieto and González-García, 2022). Whether the multidimensional construct of anxiety might influence mechanical pain sensitivity, however, is not clear. Although there is some evidence that mechanical pressure sensitivity may be related to symptoms of heightened stress in young adults (Waller et al., 2016; Waller et al., 2020), there is also evidence from animal and human studies that heightened anxiety leads to increased pain reactivity, while fear results in decreased reactivity. Racine et al. (2012), in a systematic review of 129 research articles, found the association between pressure-pain sensitivity and various measures of anxiety and depression to be largely inconsistent and contradictory with regard to their direction across outcome measures. Moreover, in the current study, we observed no significant associations between anxiety and PPTs at any site in either group. Rather curiously, however, we did observe a modest, though negative, correlation between self-reported fatigue and PPTs at the heel, bilaterally, but only in the group of runners. While there is emerging evidence that the reporting of symptoms of fatigue may be genetically linked with symptoms of negative affect, such as anxiety, and somatic complaints (Vassend et al., 2018), there is also some, albeit limited, evidence that pressure-pain thresholds beneath the PCA may be modestly reduced in healthy adults (Hedge’s g = 0.41) following a physically fatiguing, 3-h mountain trek (Barzegar et al., 2023) and that running-induced fatigue can lead to increased loading beneath the heel in rearfoot footstrikers (Hamzavi and Esmaeili, 2021).

The second point of difference between runners and non-runners in this study, was that all runners in the current study were within a “normal weight” range, while non-runners were significantly heavier, with a BMI that ranged from “normal weight” through to “Obese Class II” (WHO Expert Consultation, 2004). Overweight and obesity have been widely suggested to alter pain and somatosensory processing in humans (Walsh et al., 2018). While experimental evidence regarding pain sensitivity and adiposity is mixed (Vervullens et al., 2022), exigent research suggests that obesity is associated with heightened mechanical pain sensitivity (lowered PPTs) (Xiong et al., 2011; Tashani et al., 2017), while others have shown that greater fat free (lean) body mass is associated with lowered mechanical pain sensitivity (heightened PPTs), albeit generally in healthy, older adults (Johnson et al., 2024; Peterson et al., 2022), and in those with musculoskeletal pathology (Sylwander et al., 2021; Ferreira et al., 2022; Meert et al., 2024). Hence, it is possible that the differences observed in PPTs between runners and non-runners in the current study reflect differences in body composition between groups. It should be noted, however, that we found no significant correlations between BMI and PPT at any of the sites evaluated in either group. Moreover, as cautioned by Tashani et al. (2017) it is likely that the interaction between body composition and mechanical pain sensitivity may vary between body sites and with different stimulus types and intensities. To date, few studies have investigated the effect of body composition on mechanical pain sensitivity maps of the plantar aspect of the foot. However, it is noteworthy, that research evaluating pressure-discomfort thresholds, which might be considered a precursor to pain (Hodge et al., 2009; Johansson et al., 1999), have reported the opposite effect, in which obese individuals were found to have higher mechanical discomfort thresholds than the non-obese, but only beneath the heel, midfoot and first metatarsal head (Dueñas et al., 2021). Interestingly, the subcutaneous tissue at these sites typically demonstrates higher mechanical and electrical resistance (Frahm et al., 2013; Guo et al., 2023), and the heel and first metatarsal head are reportedly exposed to greater increases in principal stress during changes in speeds from walking to running (Mei et al., 2020; Rosenbaum et al., 1994). It is also noteworthy that in the current study, subcutaneous tissues at these same sites were significantly thicker in non-runners and were modestly, though positively, correlated with BMI (r = 0.41–0.52, P < 0.05), but only in non-runners where there was a greater range of BMI values. As noted by Finocchietti et al. (2011), a thicker superficial adipose tissue layer results in lower principal stress in deep tissue. Non-runners in the current study might, therefore, be expected to have artificially inflated PPTs at these sites, and hence, show less difference to runners than at other sites where differences in skin and thickness were less pronounced, such as the THE. As shown in Figure 2, this was not the case. Thus, while between-groups differences in the material properties of the skin and subcutaneous cannot be ruled out, we propose the hypoalgesic bias observed in runners most likely reflects a centrally mediated effect. Nonetheless, whether the widespread hypoalgesia observed in runners is best explained in terms of sensory differences or reflects a change as to what a runner considers painful requires further research. Based on the findings of the current study, future research directed toward unravelling potential relationships among body composition, psychological health, fatigue, tissue properties and mechanical pain sensitivity of the foot sole appears to be warranted.

Despite differences in the absolute magnitude of PPTs between groups, the current study observed a similar topographical pressure-pain sensitivity pattern in both runners and non-runners. Consistent with previous studies (Hodge et al., 2009; Xiong et al., 2011; Xiong et al., 2013; Weerasinghe et al., 2016; Tornero-Caballero et al., 2016; Wu et al., 2024; Ríos-León et al., 2019; Messing and Kilbom, 2001), the plantar heel (PCA) was observed to have the highest PPT in both groups, and hence the lowest sensitivity to deep pressure pain of all foot sites. Interestingly, mean PPTs observed beneath the heel of non-runners in the current study are approximately twice the peak principal stress reported beneath the heel during barefoot walking (≈250–300 kPa) at preferred speeds (≈1.0–1.3 m/s), but are only marginally higher (≈12%) than the peak stress reported beneath the heel during shod walking (≈500 kPa) (Mei et al., 2020; Wearing et al., 2009). Mean PPTs beneath the heel of runners, in contrast, are marginally lower (≈13%) than the peak stress reported beneath the heel during shod running (≈800 kPa) at preferred speed (3.0 m/s) (Mei et al., 2020). Hence, it would appear that PPTs beneath the heel are closely matched to the peak pressures that repeatedly occur with the predominant activity undertaken by each group.

In contrast to the heel, pressure-pain sensitivity was greatest for the soft tissues overlying the ABH and 1MH (Ríos-León et al., 2019). The observation of a proximal-to-distal and to a lesser extent lateral-to-medial increase in pain sensitivity across the foot sole in the current study is consistent with previous research (Ríos-León et al., 2019), and, in part, mirrors the spatial distribution reported for plantar tactile sensitivity of the foot sole in healthy adults (Dueñas et al., 2021). Spatial differences in the mechanical pain sensitivity are thought to reflect a number of peripheral factors, including the density and firing sensitivity of the deep nociceptive afferents, and the thickness and mechanical properties of the overlying plantar tissue, in addition to central pain processing mechanisms (Rodrigo et al., 2013; Vervullens et al., 2022). Although reports from microneurographic studies vary in regard to the distribution, and density of sensory afferents (Kennedy and Inglis, 2002; Corniani and Saal, 2020; Fallon et al., 2005; Strzalkowski et al., 2015b), exigent evidence currently suggests an innervation-density gradient exists across the plantar surface of the foot, which increases from proximal to distal, and to a lesser extent from medial to lateral (Kennedy and Inglis, 2002; Strzalkowski et al., 2018). While heightened innervation densities and/or excitability of mechanosensitive nociceptors are broadly believed to increase the probability of sensory activation and pain perception, differences in the thickness and mechanical properties of the skin and subcutaneous tissues are, in turn, thought to influence the mechanotransductional environment of deep tissue nociceptors (Fleckenstein et al., 2017; Strzalkowski et al., 2015a; Finocchietti et al., 2011; Melia et al., 2019).

Skin and superficial subcutaneous tissues are known to be inhomogeneous, anisotropic, and multilayered materials, which are typically in pre-stressed state, and demonstrate non-linear deformation with loading (Finocchietti et al., 2011). It is perhaps not surprising, therefore, that the relationship between PPTs and skin and superficial subcutaneous tissue thickness observed in the current study was also highly non-linear in both runners and non-runners (Figure 1). In a three-dimensional finite-element model, in which these overlaying tissues were modeled as homogeneous and hyper-elastic, Finocchietti et al. (2011) demonstrated that thicker subcutaneous tissue layers resulted in more localized and lower internal tissue stress and strain within deeper tissue and, hence, heightened PPTs. Similarly, harder skin and subcutaneous tissue layers have been shown to evoke heightened sensory and perception thresholds (Strzalkowski et al., 2015a; Strzalkowski et al., 2015b). Accurate determination of soft tissue properties, however, is challenging. Studies using standard indentometry to evaluate both PPT and tissue hardness *in vivo* have shown the PCA to be structurally stiffer than other sites of the plantar foot (Xiong et al., 2013; Weerasinghe et al., 2016; Rodrigo et al., 2013), while other studies incorporating indentation with tissue imaging approaches, have reported the opposite, with the PCA shown to have a lower material stiffness than other plantar foot tissues (Kwan et al., 2010; Chao et al., 2011; Klaesner et al., 2002). Mechanical testing of plantar tissue specimens *ex vivo* have also shown mixed results, with the material properties of the PCA reportedly higher (Ledoux and Blevins, 2007), lower (Pai and Ledoux, 2010) or no different (Pai and Ledoux, 2011) to that of other plantar tissues. Moreover, PPTs are also thought to be influenced by the mechanical properties of the deeper tissues themselves. For a given stress applied to the surface of the skin, a lower peak strain is evoked in a harder deep tissue than a softer tissue, and, as a consequence, higher pressure stimulation intensities are required to reach the strain dependent pain threshold (Finocchietti et al., 2011). Indeed, PPTs of the plantar surface of the foot have been shown to be positively correlated (r = 0.63–0.98) with indentation-based measures of secondary tissue stiffness or hardness under specified testing conditions (Xiong et al., 2013; Weerasinghe et al., 2016; Rodrigo et al., 2013). Given the obvious challenges in defining the mechanical properties of plantar tissues *in vivo*, it is interesting to note that, similar to sensitivity to light touch (Strzalkowski et al., 2015a), PPT values in both runners and non-runners in the current study could be effectively modelled as a function of the relative plantar tissue thickness, in this case using a simple logit function.

Logit functions have been widely used in value-based decision making models in marketing, economics and transportation for more than 50 years, and models decisions as a sum of weighted factors to yield the log odds (Coskunoḡl et al., 1985). More recently, the approach has also been used to describe choices related to multiple factors in both humans and animals, including sensory input and multisensory integration (Carandini, 2024). It is interesting to note that the observed bias in PPT between runners and non-runners in the current study, was primarily related to the β3 coefficient, which governs the offset in PPT, as opposed to the β1 and β2 coefficients which reflect the overall gain in PPT, and the sigmoidal shape of the curve as a function of relative tissue thickness, respectively. Although speculative, given the cross-sectional design of the current study, it is possible that the hypoalgesic offset might reflect a long-term mechanical adaptation or habituation of nociceptors to ambient stimulation levels associated with repeated foot impacts in runners. In support, animal and human studies have typically shown that net somatosensory activity is progressively reduced with repeated exposure to familiar stimuli, which is thought to aid in sensory discrimination and minimize sensitization and may be either peripherally- or centrally-mediated (Dobler et al., 2024; Barros-Zulaica et al., 2019; Klöcker et al., 2016; Graczyk et al., 2018; Brenner et al., 2000). The observations that PPTs at all test sites were heightened in runners and that normalization of PPTs of the foot to those of the hand effectively mitigated the hypoalgesic offset in runners but without influencing the shape of the PPT-tissue-thickness function, as denoted by β2 coefficients, further suggests that centrally-mediated processes may prevail in runners over differences in peripheral factors related to the structural and mechanical properties of the skin and subcutaneous tissue, *per se*. Moreover, it would appear that any centrally- or peripherally-mediated effect that may account for the bias in pain sensitivity between groups, does not substantively alter the spatial distribution pattern of PPTs across the plantar aspect of the foot. It is also worth noting that β2 coefficients in this study were always within 50 microns of measures of skin and subcutaneous tissue thickness at the PCA, which falls well within expected measurement error of sonographic-based measures of tissue thickness, and suggests that the material properties of plantar tissue across foot sites were relatively uniform.

Consistent with previous research, we also observed that the THE of the palmar hand was more sensitive to mechanical pain than the plantar foot (Rolke et al., 2005), with PPTs ranging from 40% to 80% of those of the plantar foot; presumably, reflecting its lower tissue thickness and greater innervation density (Kennedy and Inglis, 2002; Strzalkowski et al., 2018). While the models demonstrated adequate fit for each limb, it should be noted that normalization not only lowered the overall range of PPT values in runners but also reduced the overall accuracy of the fit, suggesting that there are minor site-specific differences between palmar and plantar foot innervation in runners and non-runners. We specifically normalized plantar foot values to those of the THE given its ease of accessibility, its remote, non-weight-bearing location and that the thickness of the skin and subcutaneous tissue at the site is minimal but also comparable between groups. Although such remote sites are commonly evaluated clinically, to aid in the evaluation of peripheral versus centralized pain (Eckenrode et al., 2019), their use in the normalization of PPT values has received comparatively little scientific attention. Of the few studies that have been undertaken, normalization of PPT values to remote sites tended to show greater sensitivity and less temporal drift over the short term than absolute values and greater stability over time (Rolke et al., 2005; Kosek et al., 1993; Fredriksson et al., 2000). Here, we show that it may also be a useful and relatively quick method for ensuring homogenous comparator groups in case-control studies in which comparisons in pressure-pain sensitivity of the plantar foot are planned.

As with all research, this study has a number of limitations and delimitations. Chiefly, while the cross-sectional nature of the current study effectively allowed for the evaluation of differences in topographic pressure sensitivity of the plantar foot in runners and non-runners, the design does not allow for conclusions regarding potential cause-and-effect to be made. Thus, it is unknown whether the bilateral hypoalgesia observed at the sole of the feet of runners in this study reflects an adaptive response to endurance running or whether it preceded the uptake of endurance running in the study cohort. Similarly, this study did not quantify the running history and training loads of runners in detail, nor measure all of the physiological and psychological factors that have been suggested to influence PPTs in athletes (Lemley et al., 2015). Moreover, the current study specifically explored the role of relative skin and subcutaneous tissue thickness rather than the material properties of these tissues, given challenges associated with the accurate quantification of soft tissue properties *in vivo* and the current limitations associated with the use of elastographic and indentation-based methods to estimate the viscoelastic properties of tissues (Chatzistergos et al., 2022; Oddes and Solav, 2023; Wearing et al., 2024). Given the current findings, however, it is recommended that future prospective research evaluate the temporal relationship of topographical pressure sensitivity maps of the hands and feet with estimates of running history, training load, psycho-physiological factors, and the relative thickness and material stiffness of the plantar tissues in trained, endurance runners and non-runners as well as in endurance athletes involved in largely non-weightbearing sports, such as swimming.

In conclusion, the current study has demonstrated that, despite having thinner plantar soft tissues than healthy non-runners, endurance runners demonstrate systematically higher PPTs beneath all sites of the foot, and at the thenar eminence of the hand. The hypoalgesic bias in plantar foot PPTs in active runners and topographical variation in PPTs observed across the plantar aspect of the foot in both groups can be effectively modelled as a function of relative plantar tissue thickness, using a simple three-component logit model. Moreover we show, for the first time, that the hypoalgesic bias in runners may be mitigated through the normalization of pedal PPT values to those of the thenar eminence of the hand, without appreciably altering the overall shape of the logit function.

## Data Availability

The raw data supporting the conclusions of this article will be made available by the authors, without undue reservation.
